# Editorials

**Published:** 1893-01

**Authors:** 


### Editorial.

### PRELIMINARY ANNOUNCEMENT.

The following is a part of the special program of the sixth annual meeting of the National Association of Railway Surgeons, embracing the United States of America, the Dominion of Canada and the Republic of Mexico, to be held at Omaha, Neb., the last Wednesday, Thursday and Friday of May, 1893.

General subject: Injuries of the Cord and its Envelopes Without Fracture of the Spine.

1st, History, by Dr. Geo. Ross, Chief Surgeon Richmond & Danville R. R., Richmond, Va.

2nd, Anatomical Landmarks, by Dr. Jabez N. Jackson, Surgeon Wabash R. R., Kansas City, Mo.

3rd, Physiology of the Spinal Cord, by. Dr A. P. Grinnell, Chief Surgeon Central Vermont R. R., Burlington, Vt.

4th, Experimental Research, by Dr. B. A. Watson, Surgeon Pennsylvania R. R., Jersey City, N. J.

5th, An Experimental Study of Spinal Myelitis and Minengitis, by Dr. Geo. A. Baxter, Div. Surg. Chattanooga Southern R. R., Chattanooga, Tenn.

6th, The Clinical Aspects of Spinal Localization, by Dr. Nicholas Senn, Surgeon Chicago, St. Paul & Kansas City R. R., Chicago, Ill.

7th, Diagnosis from the Standpoint of the Neurologist, by Dr. C. P. Hughes, Consulting Surgeon Missouri Pacific R. R., St. Louis, Mo.

8th. Pathology and Pathological Anatomy, by Dr. Samuel C. Benedict, Surgeon Richmond & Danville R. R., Athens, Ga.

9th, Prognosis, by Dr. Samuel S. Thorn, Chief Surgeon Toledo, St. Louis & Kansas City R. R., Toledo, Ohio.

10th, Treatment, by Dr. W. B. Outten, Chief Surgeon Missouri Pacific R. R., St. Louis, Mo.

11th, Medico-Legal Aspects, by Judge J. H. Collins, Chief Counsel Balto. & Ohio R. R., West of the Ohio river, Columbus, Ohio.

12th, Statistics of the Amount of Money paid by the Railroads of the United States, During the last Ten Years, for Alleged Injuries of the Spine, by Dr. F. K. Ainsworth, Surgeon Southern Pacific R. R., Los Angeles, California.

13th, Clinical Report—1st, From a Medical Aspect—(a) Permanent Injuries—(b) Alleged Injuries. 2nd, From a Legal Aspect—(a)Settled with Suit—(b) Settled without Suit—(c) Miscellaneous, by Dr. Geo. Chaffee, Surgeon Long Island R. R., Brooklyn, N. Y.

C. W. P. BROCK, M. D., Pres't, Richmond, Va.
E. R. LEWIS, M. D., Sec'y, Kansas City, Mo.

---

Dr. Virgil O. Hardon, of Atlanta, was married December 27th, to Miss Bertha Wardell, of Bainbridge, Ga. The ceremony was performed at the Episcopal Church of the latter city. Dr. M. Ashby Purse, of Atlanta, acted as best man. The RECORD extends congratulations.

### RAILWAY SURGERY AT THE PAN-AMERICAN MEDICAL CONGRESS.

A Section of Railway Surgery of the Pan-American Medical Congress has been organized with Dr. C. W. P. Brock, of Richmond, Virginia, as executive president. A full list of officers has been provided for each of the constituent countries. At the eleventh annual meeting of the Wabash Railway Surgical Association—the first organization of the kind—Dr. C. B. Stemen, of Fort Wayne, was by unanimous resolution requested to prepare a paper on "Organized Railway Surgery," and read the same before the Section on Railway Surgery of the Pan-American Medical Congress. At the same meeting, Dr. Hal C. Wyman, of Detroit, offered the following, which was unanimously adopted:

Resolved that each member of this Association solicit his Congressman to interest himself in legislation in favor of the Pan-American Medical Congress.

---

A post-graduate medical school for clinical instruction has been organized in Atlanta, Ga., to be known by the name of Atlanta Polyclinic. Its faculty is composed of a number of live, energetic, active physicians who are determined to make the Polyclinic a success in every particular. It is a laudable enterprize and should have the encouragement and support of every member of the profession in the South, as it is the only institution of that character in the South that will be open all the year to physicians and graduates of medicine for clinical instruction. They have procured a building well adapted to clinical teaching, giving hospital advantages in connection with the school. Polyclinic opens March 15, 1893. Regular "ad" will appear in next issue.

---

At the meeting of the Atlanta Society of Medicine, December 20th, the following officers were elected for the ensuing year: Dr. W. S. Elkin, President, Dr. J. W. Duncan, Vice-President, Dr. M. B. Hutchins, Secretary, Dr. L. B. Grandy, Treasurer, and Dr. N O. Harris, Librarian and Corresponding Secretary.

We desire to call our readers' attention to the following new advertisements, also to all others—you will find that they are reliable and the best in the country:

Johnson & Johnson, Parke, Davis & Co., Coulter Vaporizer Co., C. N. Crittenton, California Fig and Syrup Co., Odd Chem. Co., Dios Chemical Co., New York Medical Journal, Hopkins & Atkins, The Nurse, Whitman Saddle Co., The Advertiser, Anticlavus Chemical Co., The Gasconade, Reed & Carnrick, Robinson Pettit Co., The Natural Body Brace Co., The Hall Capsule Co., Cincinnati Medical Journal. We hope all of our readers and patrons a happy and prosperous new year.

---

The Winyah Sanitarium, Asheville, N. C., is being remodled and equipped in the most approved fashion, and when completed will be up to the most recent advances made in institutions for the treatment of throat and lung diseases. Dr. Karl von Ruck expects to open the sanitarium for the reception of patients about November 20th, 1893. From the establishment of the institution until the burning of same some weeks ago, its success has been phenomenal, and it is hoped that it may secure the liberal patronage by the profession that has formerly characterized it.

---

The Jackson County Medical Society of Alabama held their eleventh annual meeting in the county court room, at Scottsboro, Ala., January 3. On the same night they were tendered a banquet at the Hotel Harris.

---

Dr. I. O. B. Bush, of Colquit, Ga., was married to Miss Leila McNaw, December 23d, 1892.

---

We hope all that are due us for subscription will remit at once.

---

Read premium list.

